# Notes and Notices

**Published:** 1893-06

**Authors:** 


### NOTES AND NOTICES.

Childhood.—" A score of years' experience," says Dr. George William Winterburn, in the December *Childhood*, " in dealing with the ailments of children, and constant association with them, has impressed the thought most deeply that children suffer constantly from the unwisdom of their elders. This lack of wisdom in child-culture is unperceived by most parents and teachers. The average parent desires to do well by his child, and truly believes he does as well as circumstances permit. It is true he has never made any particular study of his duties as a parent. He has a vague sense that such knowledge comes by intuition. To suggest to him that he should prepare for parenthood with the same earnestness of purpose and the same willingness to learn from those who have given special attention to this subject, as he would in fitting himself for any other profession or occupation, seems to him absurd."

The Libby Prison War Museum.—Of the many attractions outside of the World's Fair in Chicago, there are but few in which there is so much interest centered as there is in the Libby Prison War Museum. In 1889 this celebrated prison was removed from Richmond to Chicago and converted into a War Museum. The project was undertaken by a syndicate of the best known business men of the city whose enterprise was conceived in a commercial spirit, but has attained a national reputation. A project such as this was never before heard of. To move a brick and stone building the size of Libby more than a thousand miles, across rivers and mountains, was an enterprise than many of the best known contractors in the West refused to undertake at any price. But the move was made with success. Then the famous old structure was filled with war material that represents the work of a lifetime and the expenditure of half a million dollars. The great collection is conceded to be second to none in the country and includes much of the most valuable material that the greatest civil war the world has ever known has left to posterity. The collection includes thousands and thousands of relics of every description, many of which form important links in the history of the nation. The old building itself is fraught with interesting memories, and the story of the celebrated tunnel escape of February 19th, 1864, never fails to interest the visitors. One hundred and nine Union officers made their escape through that tunnel, which formed one of the most thrilling events in the history of the war.

Shall Hahnemann's "Chronic Diseases" be Reprinted?—"Some one ought to reprint Hahnemann's *Chronic Diseases*." So say many practitioners. But to bring out such a work involves the expenditure of a goodly sum of money—a risky expense that few publishers care to assume. Messrs. Bœricke & Tafel have, however, determined to make the attempt to reprint this grand old work. Estimates have been obtained, the cost figured out, and now it only remains for the gentlemen of the homœopathic medical profession to indicate their wishes. If a sufficient number will subscribe to the undertaking to enable the publishers to see their way toward paying for paper and type-setting, the old book will again be obtainable; otherwise it will remain out of print.

Subscribe through your regular pharmacist, or book-dealer, or direct to the publishers, Bœricke & Tafel, 1011 Arch Street, Philadelphia, Pa.

Free Trip to Chicago.—Any one of our readers who desire a free trip to Chicago to see the great exposition should consult the advertisement of the Scott Seed Company on advertising page 3.

The Yale Chair.—The value and convenience of this chair cannot be appreciated until one is in a predicament where the ingenious variations in its structure can be most advantageously applied if it be at hand to relieve one of the difficulty.

---

### FUN FOR DOCTORS.

Mrs. Youngquack—"You are so persevering and hopeful, dear; you remind me of patience sitting on a monument."

Dr. Youngquack—"I feel blue enough to remind you of the monuments sitting on my patients."

Nurse—"How am I to treat that little tailor who was brought to the hospital to-day? He's terrible thin, you know."

Physician—"Put two mustard plasters on him—one on his chest and one on his back."

Nurse—"That's all right, but suppose the two plasters come together?"—*Fliegende Blaetter.*

Her Very Own.—Dyspepsia specialist (irritably)—"But, Madame, you must chew your food. What were your teeth given you for?"

Female patient (calmly)—"They weren't given to me. I bought 'em."—*Life.*

---

Customer (in great haste)—"Give me some Soothing Syrup, quick!"

Leisurely Druggist—"Will you have a four-ounce or an eight-ounce bottle?"

Customer—"Oh! I want a keg of it, it's twins!"

